# Warm Water Bath Stimulates Phase-Shifts of the Peripheral Circadian Clocks in PER2::LUCIFERASE Mouse

**DOI:** 10.1371/journal.pone.0100272

**Published:** 2014-06-16

**Authors:** Nobuaki Ohnishi, Yu Tahara, Daisuke Kuriki, Atsushi Haraguchi, Shigenobu Shibata

**Affiliations:** Laboratory of Physiology and Pharmacology, School of Advanced Science and Engineering, Waseda University, Tokyo, Japan; Nagoya Universitym, Japan

## Abstract

Circadian clocks in the peripheral tissues of mice are known to be entrained by pulse stimuli such as restricted feeding, novel wheel running, and several other agents. However, there are no reports on high temperature pulse-mediated entrainment on the phase-shift of peripheral clocks *in vivo*. Here we show that temperature treatment of mice for two days at 41°C, instead of 37°C, for 1–2 h during the inactive period, using a temperature controlled water bath stimulated phase-advance of peripheral clocks in the kidney, liver, and submandibular gland of PER2::LUCIFERASE mice. On the other hand, treatment for 2 days at 35°C ambient room temperature for 2 h did not cause a phase-advance. Maintenance of mice at 41°C in a water bath, sustained the core body temperature at 40–41°C. However, the use of 37°C water bath or the 35°C ambient room temperature elevated the core body temperature to 38.5°C, suggesting that at least a core body temperature of 40–41°C is necessary to cause phase-advance under light-dark cycle conditions. The temperature pulse stimulation at 41°C, instead of 37°C water bath for 2 h led to the elevated expression of *Per1* and *Hsp70* in the peripheral tissue of mice. In summary, the present study demonstrates that transient high temperature pulse using water bath during daytime causes phase-advance in mouse peripheral clocks *in vivo.* The present results suggest that hot water bath may affect the phase of peripheral clocks.

## Introduction

The circadian clock enables an organism to adapt its physiology and behavior to routine environmental changes. Circadian clock system is located in the suprachiasmatic nucleus (SCN) in the brain, which is the main oscillator, as well as in the peripheral organs, the peripheral oscillator. Genetic and molecular approaches have identified a core clock mechanism of the circadian oscillator that is governed by interconnected transcriptional and translational delayed feedback loops [1], [2]. The CLOCK/BMAL1 heterodimer initiates the transcription process by binding to specific DNA elements, E- and E'-boxes in the promoters of target genes [3], [4]. This set of activated genes includes members of the negative limb of the feedback loop including the *Per* (*Per1* and *Per2*) and *Cry* (*Cry1* and *Cry2*) genes [3], [5], [6]. The resulting PER and CRY proteins dimerize and inhibit further CLOCK/BMAL1 transcriptional activity, allowing the cycle to repeat from the level of low transcriptional activity [7]–[9]. The circadian clock genes are necessary for daily rhythm of core body temperature and also for cold temperature-induced heat production behavior. Mutant mice with *Rev-erbα* gene exhibit resistance to body temperature decrease during the inactive period compared to the wild type mice [10]. On the other hand, mutant mice with *Per2* gene exhibit augmented body temperature and are not viable in decreased ambient temperature [11].

In *in vitro* cell line experiments, NIH3T3 cells and RAT1 fibroblasts showed an entrained circadian rhythm at temperatures within the physiologic range (36°C to 38.5°C) or in routine temperature differences of only 3°C [12], [13]. In *in vitro* SCN and peripheral tissues experiments, circadian changes in temperature comparable to those seen with core body temperature rhythms are capable of entraining and enhancing the amplitude of the circadian rhythms of peripheral tissues, while the adult SCN remains resistant [14]. The temperature pulse-mediated resetting of *Per2* expression in tissue explants has been shown to be blunted in the presence of the heat shock transcription factor (HSF) inhibitor [14] or in HSF1-deficient fibroblasts [15]. Thus, HSF may be involved in the temperature-induced phase entrainment of circadian clocks [12], [14]. Recently, the cold-inducible RNA-binding protein (Cirp) mRNA and protein levels oscillated throughout the day in cells has been reported to be synchronized with body temperature rhythms (34–38°C), but not in cells maintained at 36.5°C, after synchronization of core clock machinery by serum stimulation. The reduction of CIRP protein levels in fibroblasts by RNA interference showed that CIRP is required for high-amplitude circadian gene expression [16].

Very few *in vivo* studies have investigated temperature cycle as an entrainment factor in endothermic animals such as birds and mammals. Occasional studies on a few species of rodents [17]–[21] have indicated that temperature cycles have the potential to entrain circadian rhythms, but are rather weak entraining factors. Almost all experiments thus far evaluated the locomotor activity as a function of the circadian rhythm, which is controlled by SCN circadian clocks. Daily cycles of light and temperature are perhaps the two most reliable environmental time cues for living systems on earth. Although it is well established that light is the strongest time cue for the mammalian SCN circadian rhythm, mechanisms underlying the entrainment of peripheral circadian clocks in response to high temperature pulses has not been well established *in vivo*.

Recently, we established a simple experimental protocol to evaluate the circadian clock rhythm of peripheral organs with an *in vivo* imaging system (IVIS) using PER2::LUCIFERASE (PER2::LUC) knock-in mice [22]. In the present experiment, we examined whether circadian rhythm of peripheral clocks can be entrained in response to warm temperature pulse using the IVIS method. Due to the compensatory response in mice, ambient room temperature fluctuations (24–35°C) may be insufficient to continuously increase the core body temperature in mice. Hence, in the present experiment, mice were exposed to warm water bath to ensure the continuous maintenance of elevated core body temperature.

## Methods

### Ethics Statement

All experimental protocols were approved by Committee for Animal Experimentation of the School of Science and Engineering at Waseda University (permission protocol #; 2013-A058, 2013-A061) and in accordance with the law (No. 105) passed and notification (No. 6) of the Japanese Government.

### Animals

PER2::LUC C57BL/6J knock-in mice (courtesy of Dr. Joseph Takahashi, Southwestern Medical Center, Texas University, TX, USA) [23], were backcrossed more than 5 times against ICR mice (Tokyo Laboratory Animals, Tokyo, Japan). Heterozygous PER2::LUC knock-in mice were kept in a room maintained on a 12∶12 h light-dark cycle with lights on at 08∶00, which we defined as a zeitgeber time 0 (ZT 0), at a temperature of 23±1°C, 60±5% humidity, and a light intensity of 100–150 lux at cage level, and were provided with a standard monounsaturated fatty diet (Oriental Yeast Co. Ltd., Tokyo, Japan) and water *ad libitum*. Four female mice were housed as a single group for the current experiments. In addition, the experiment was conducted on a separate set of male mice to confirm that the phase change was due to the high temperature of the water bath and not the estrous cycle seen in female mice.

### Exposing mice to temperature-controlled water bath

Water incubator and mixer (As one Inc., Osaka, Japan) were set at the edge of case and a cylindrical sieve made of 6.7 mm square stainless mesh was placed inside. Water was poured into the case to attain a height of 26–28 mm from the surface of the water to the stainless net. After the incubator was set to maintain the required temperature, a plastic pipe whose diameter was 131 mm, height was 300 mm, was set on the cylindrical sieve. Warm water was poured for experimental mice, but no water for control mice, and intact mice were kept under home cage. Two mice were placed on the cylindrical sieve during treatment (0.5–2.0 h) (Fig. S1A) once a day or once a day for 2–3 continuous days. In some mice, warm water treatment was applied for 2 continuous days, and then bioluminescence recording was started 24 h after heating. Non-photic entrainment stimuli such as food and novel wheel running were provided mid-day to stimulate the phase-advance of peripheral clocks [24]–[26]. Therefore, in the present experiments, mice were subjected to warm water stimulation at mid-day.

### Measurement of the core body temperature

We prepared PER2::LUC heterozygous mice for recording their core body temperature. A small temperature sensor (Thermochron SL; KN Laboratories Inc., Osaka, Japan) was shaped similar to a button battery. One side of the sensor was covered with a plastic shield and the other side served as the sensing surface. The mice were anesthetized with midazolam/xylazine. The sensor was then implanted, with the sensing surface near the liver, and the covered surface just under the skin. This specific positioning prevented the sensor from detecting changes in the environmental temperature. After a recovery period of one week, the temperature was automatically recorded at 10 min intervals for one month. The recordings were analyzed using RhManeger (Version 2.10; KN Laboratories Inc., Osaka, Japan).

### *In vivo* monitoring of PER2::LUC bioluminescence

*In vivo* monitoring of PER2::LUC bioluminescence was performed as previously described [22] using an IVIS kinetics system (Caliper Life Sciences, Hopkinton, MA, USA, and Summit Pharmaceuticals International Corporation, Tokyo, Japan). Mice were anaesthetized with isoflurane (Mylan Inc., Tokyo, Japan) and concentrated oxygen (SO-005B; Sanyo Electronic Industries Co. Ltd, Okayama, Japan) in a black box using the gas anesthesia system (XGI-8; Caliper Life Sciences). Anesthetized mice were injected with d-luciferin potassium salt (Promega, Madison, WI, USA) subcutaneously (15 mg/kg). Images were acquired with an exposure time 1, 6, and 8 min after luciferin injection in the dorsal-up position for the kidney, and at 10 and 12 min after injection in the ventral-up position for the liver and the submandibular gland. Each bioluminescence image was merged with the corresponding grey-scale image.

Images were obtained 6 times a day (ZT 7, 11, 15, 19, 23, and 3) (Fig. S1B). Mice were returned to their home cages after each imaging session, where they recovered quickly from anesthesia. The total time under anesthesia was approximately 20 min per session. A previous study has shown that luciferase activity in the peripheral tissues and behavior were unaffected by four 1-h-long sessions of anesthesia and bioluminescence analysis per day [22].

### Analysis of *in vivo* monitoring data

**Figure 1:**
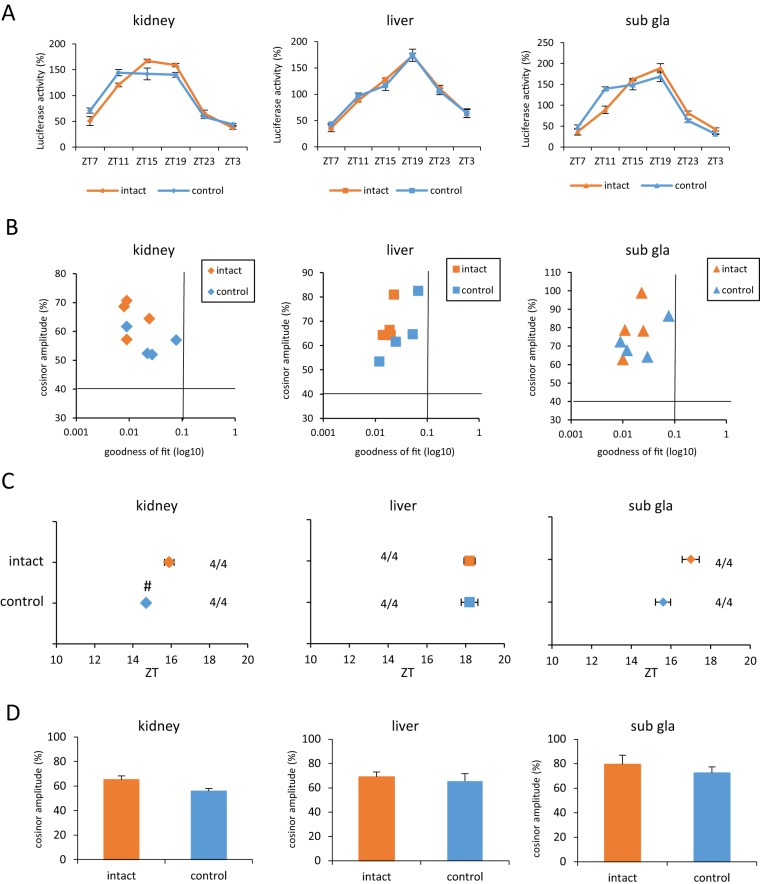
The control group did not exhibit peripheral clock phase-shift. (A) Waveform of PER2::LUC expression in various tissues. (B) Correlation maps of PER2::LUC rhythms between cosinor amplitude and index of goodness of fit. The dashed line indicates 40% of cosinor amplitude and 0.1 of goodness of fit. (C) Average of PER2::LUC expression in peak phases for each organ in each group. Numbers next to plots indicate the rates of mice whose PER2::LUC expression was rhythmic (rhythmic mice numbers/total mice numbers). (D) Average cosinor amplitude for each organ in each group. All of the values are expressed as mean ± or + SEM except (B). # p<0.05 versus intact by Mann-Whitney test.

*In vivo* monitoring data were analyzed as described previously [22]. The bioluminescence emitted from each organ (kidney, liver, and submandibular gland) was calculated automatically using Living Image 3.2 software (Caliper Life Sciences). For each organ, the region of interest was set to the same shape and size in all images. In case of the kidney, data from the right and left kidneys were combined for the analysis. The average photon/sec value from the set of 6 time points for each day was taken as 100%, and the bioluminescence rhythm for the entire day was expressed as a percentage of each set for each organ. The peak phase and amplitude of the normalized data were determined using the single cosinor procedure (Acro.exe, version 3.5; [27]). Accuracy of rhythmicity was evaluated by cosinor amplitude and goodness of fit, and peripheral clock oscillation with <40% in amplitude and/or 0.1> in goodness of fit was judged as arrhythmicity. As we did not find any cases in intact mice and control mice (Fig. 1B), we followed the guidelines from our previous paper [22].

### Isolation of total RNA and Real-time RT-PCR

The messenger RNA from tissues was measured by real-time reverse transcription polymerase chain reaction (RT-PCR), as described previously [28], [29]. A total RNA concentration of 15 ng was reverse transcribed and amplified with One-Step SYBR RT-PCR kit (TaKaRa Bio Inc. Shiga, Japan) in Piko Reall (Thermo Fisher Scientific Inc. Kanagawa, Japan). Specific primer pairs were designed based on published data for *Gapdh*, *Per1*, *Per2*, *Bmal1*, and *HSP70* genes. The primer sequences were as follows: mouse *Gapdh* 5′-tggtgaaggtcggtgtgaac-3′, 5′-aatgaaggggtcgttgatgg-3′; mouse *Per1*: 5′-caagtggcaatgagtccaacg-3′, 5′-cgaagtttgagctcccgaagtg-3′; mouse *Per2*: 5′-tgtgtgcttacacgggtgtccta-3′, 5′-acgtttggtttgcgcatgaa-3′; mouse *Bmal1*: 5′-ccacctcagagccattgataca-3′, 5′-gagcaggtttagttccactttgtct-3′; mouse *Hsp70*: 5′- aacgtgctgcggatcatcaa-3′, 5′-gaagrccrgcagcagcttct-3′. The relative levels of the target gene PCR products were normalized to those of *Gapdh*. Data were analyzed using the delta-delta Ct method. A melt curve analysis was performed to check for non-specific products. The results indicated the absence of nonspecific products.

### Statistical analysis

All values are expressed as mean + or ± SEM. Statistical analyses was performed using GraphPad Prism version 6.03 (GraphPad software, San Diego, CA, USA). We checked if the data were distributed normally by using the D'Agostino-Pearson/Kolmogorov-Smirnov test. In addition, we checked for equal or biased variation using the F-value test. Parametric analysis for multigroups was conducted using a one-way ANOVA with a Tukey test for post hoc analysis, and non-parametrical analysis for multigroups was done using the Kruskal-Wallis test with a Dunns test for post hoc analysis. The Mann-Whitney test was used for non-parametrical analysis of 2 groups.

## Results

### Temperature pulse of 41°C for 2 h causes a phase-shift in the peripheral circadian clocks

We used a temperature controlled water bath and placed the mice to change the core body temperature of the mice efficiently. Before examining the effect of temperature pulse using water baths, we checked the difference between the intact group and the control group (Fig. 1). The control group represented a group of mice placed in a cylindrical sieve at room temperature without water. Waveforms of bioluminescence rhythm of kidney, liver, and submandibular gland (sub gla) recorded in the intact or the control mice are shown in Figure 1A. We judged whether luciferase activity was rhythmic by checking whether they satisfied followed criteria: cosinor amplitude >40% and goodness of fit <0.1 (Fig. 1B) according to the previously described method [22]. The intact and the control group mice exhibited rhythmic luciferase activity. The PER2::LUC expression phases of peripheral clock were slightly advanced in the control group than in the intact group (Figs. 1A, C). There were no differences in cosinor amplitude between two groups (Fig. 1D).

**Figure 2:**
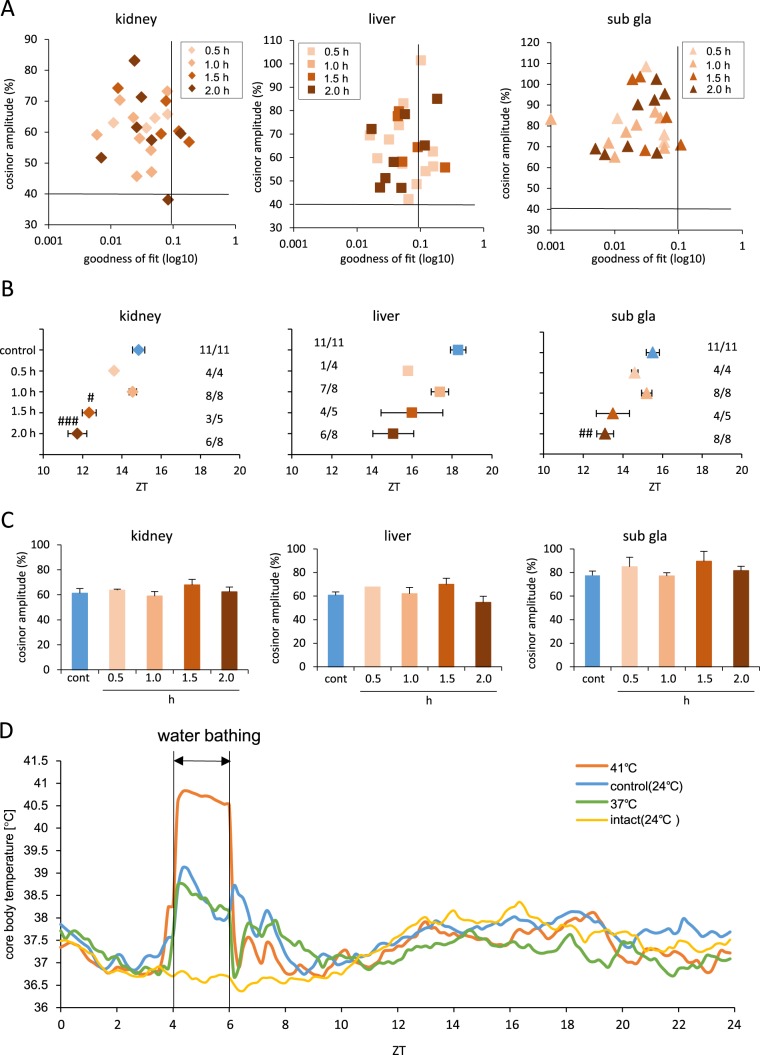
Exposing mice to 41°C water bath advanced the phase in a time-dependent manner. (A) Correlation maps of PER2::LUC rhythms between cosinor amplitude and index of goodness of fit. The dashed line indicates 40% of cosinor amplitude and 0.1 of goodness of fit. (B) Average of PER2::LUC expression peak phases for each organ in each group. Numbers next to plots indicate the rates of mice whose PER2::LUC expression was rhythmic. #p<0.05, ##p<0.01, ###p<0.001 vs. control by Dunn test. Statistical analysis was not applied for the liver in figure B because of N = 1 in the 0.5 h group. (C) Average cosinor amplitude for each organ in each group. Graphs using data from mice whose PER2::LUC expression was rhythmic. (D) Average core body temperature for each group during day 1, n = 8 for 37°C and n = 16 for 41°C, control, and intact groups. All values are expressed as mean ± or + SEM except (A) and (D).

Next, we examined the effect of 41°C heat shock using the 41°C water bath on the peripheral clock phase-shift and if it was dependent on the duration of the pulse stimulation (Fig. 2). We have presented each set of data for judgment of oscillation accuracy (Fig. 2A). Of the 25 water bath samples, four, seven, and a single sample from the kidney, liver, and the sub gla were omitted from data analysis (Fig. 2B). The phase-shift of the peripheral clock was dependent and proportional to the duration of the stimulus (Fig. 2B). The waveform of the liver clock in mice that were kept in the 41°C water bath for 1.5 h or 2 h peaked at ZT11 and at ZT19 as two peaks. However, that of the kidney and sub gla peaked at ZT11 as single peak (Fig. S2). Since two desynchronized waveforms with a single peak could have been observed from any subdivision of the liver, we analyzed the bioluminescence rhythm in 12 subdivisions from one liver sample (Figures S3A and B). However, waveforms with two peaks for the total liver still showed bimodal peaks in each subdivision (Figure S3B), but those with one peak for the total liver showed a single peak in each subdivision (Figure S3A). There were no differences detected in cosinor amplitude in any of the organs among the five groups (Fig. 2C). To record the accurate core body temperature that acts as a stimulus for phase-shift in the peripheral organs, we installed a small sensor in the intraperitoneal cavity of the experimental mice. Core body temperatures in the group treated using the 41°C water bath were maintained at 40–41°C during ZT 4–6 (Fig. 2 D).

The body temperature of female mice fluctuates with the estrous cycle, and these changes possibly influence the warm water–induced phase reset seen in female mice. To confirm that warm water causes the circadian phase reset, the experiment was conducted separately on male mice. These mice that were kept in 41°C water bath for 2 h over 2 days exhibited clear and significant phase advances of all peripheral clocks (Fig. S4).

**Figure 3:**
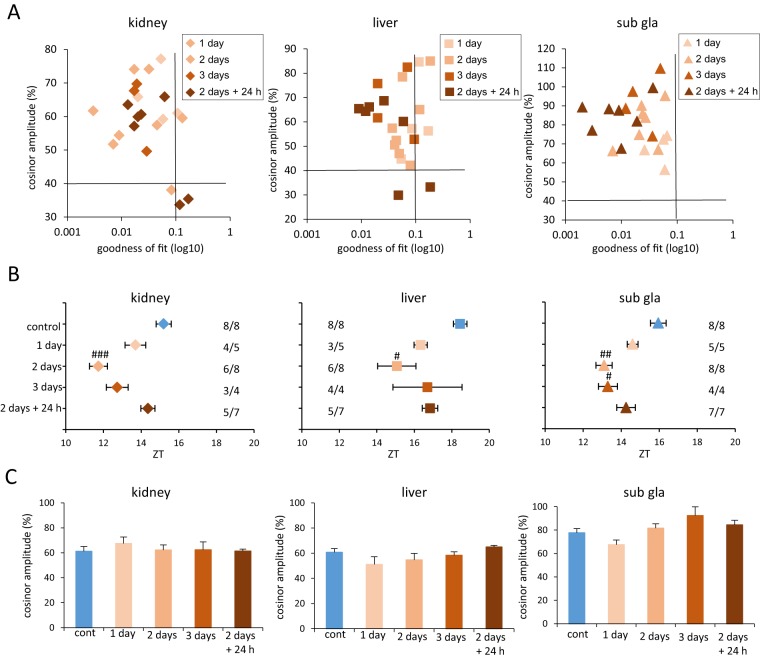
Temperature treatment at 41°C advanced the phase depending on the number of days. (A) Correlation maps of PER2::LUC rhythms between cosinor amplitude and index of goodness of fit. The dashed line indicates 40% of cosinor amplitude and 0.1 of goodness of fit. (B) Averaged PER2::LUC peak phases of each organ for each group. Numbers next to plots indicate the rates of mice whose PER2::LUC expression was rhythmic. #p<0.05, ## p<0.01, ###p<0.001 versus control mice by Dunn test. (C) Average cosinor amplitude of each organ for each group. Graphs constructed using data from mice whose PER2::LUC expression was rhythmic. All values are expressed as mean ± or + SEM except (A).

In the next experiment, we determined whether placing mice in a 41°C water bath resulted in advanced phase-shift in each organ and was dependent on the duration of treatment (1 day, 2 days, and 3 days) (Fig. 3, Fig. S5). Of the 17 samples, four kidney and four liver samples were omitted from data analysis and no samples were excluded for sub gla (Fig. 3B). A two-day treatment schedule consisting of a single 2 h session per day was sufficient to stimulate a phase-advance of the peripheral clocks (Fig. 3B). The 3-day treatment schedule caused a smaller phase-advance of the kidney and liver clocks than the 2-day treatment, but the phase advance of the sub gla clock in both treatment schedules were similar (Fig. 3B). The waveform of the liver clock in the 3-day treatment schedule peaked once (Fig. S5), while that in the 2-day treatment peaked twice. We then confirmed whether the phase-advance caused by the water bath treatment of 2 h at 41°C and over 2 days was a transient or permanent phase shift. In this experiment, we examined whether the phase-advance caused by the water bath treatment at 41°C was maintained or pull-backed to the control phase. This was studied via an IVIS recording that was done 24 h after the final heating. The phases of the peripheral clocks, especially the kidney clock, returned to the control level 24 h after heating (Fig. 3B). There were no differences in cosinor amplitude among the five groups in any of the organs tested (Fig. 3C).

**Figure 4:**
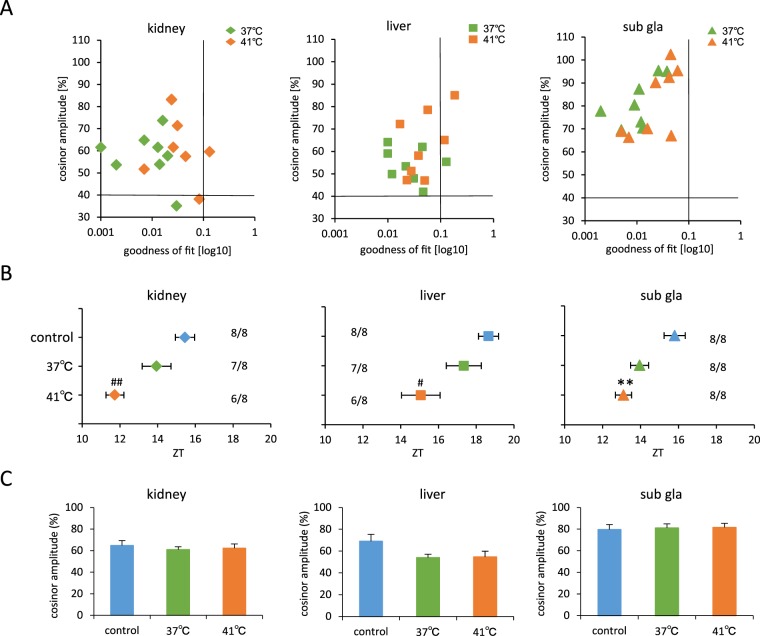
Exposing mice to 41°C or 37°C water bath stimulated phase-advance. (A) Correlation maps of PER2::LUC rhythms between cosinor amplitude and index of goodness of fit. The dashed line indicates 40% of cosinor amplitude and 0.1 of goodness of fit. (B) Average PER2::LUC peak phases of each organ for each group. Numbers next to plots indicate the rates of mice whose PER2::LUC expression was rhythmic. #*p*<0.05, ##*p*<0.01 versus control by Tukey test. **p<0.01 versus control by Dunn test (C) Average cosinor amplitude of each organ for each group. Graphs using data from mice whose PER2::LUC expression was rhythmic. All values are expressed as mean ± or + SEM except (A).

We examined the effect of different temperature pulse in stimulating the phase-shift (Fig. 4, Fig. S6). Of the 16 samples, three kidney and three liver samples were omitted from data analysis, and none was excluded for the sub gla (Fig. 4B). Maintenance of mice in the water bath set at 41°C led to a significant phase-advance of the peripheral clock in the kidney, liver, and sub gla compared with the corresponding organs of mice maintained at 37°C and those of the control group (Fig. 4B). There were no differences in cosinor amplitude among the three groups (Fig. 4C). The core body temperature of mice treated at 37°C was found to be elevated to 38–39°C, although this change was very similar to that observed in control mice (Fig. 2D).

**Figure 5:**
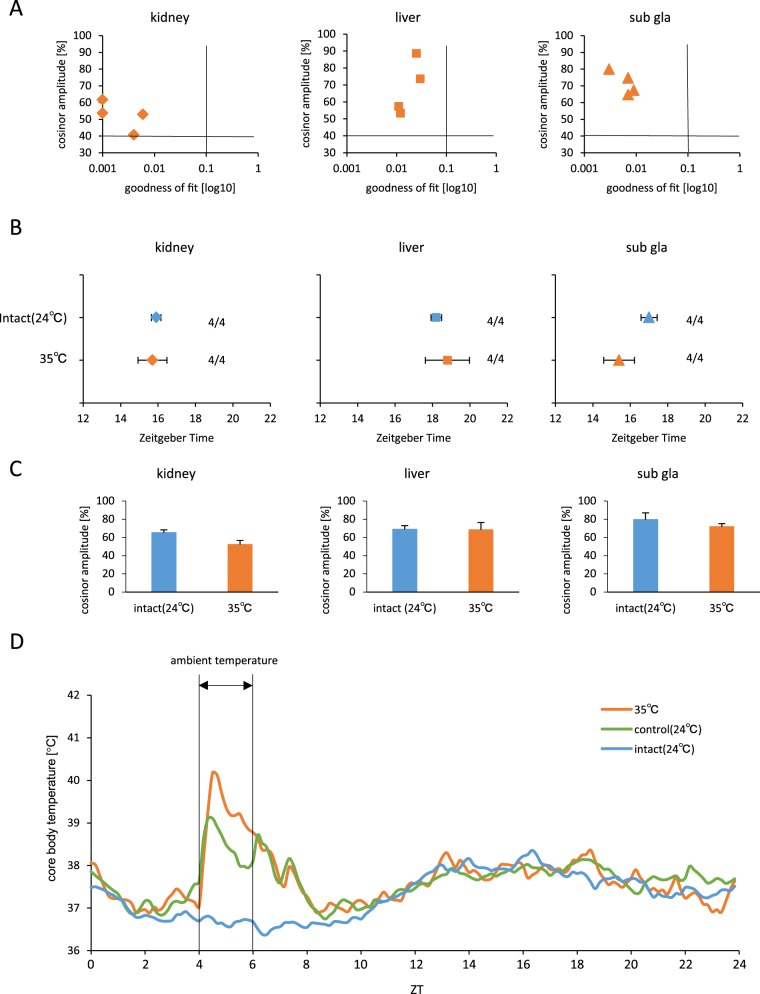
Maintenance of mice at 35°C ambient temperature room did not shift the phase. (A) Correlation maps of PER2::LUC rhythms between cosinor amplitude and index of goodness of fit. The dashed line indicates 40% of cosinor amplitude and 0.1 of goodness of fit. (B) Average PER2::LUC peak phases of each organ for each group. Numbers next to plots indicate the rates of mice whose PER2::LUC expression was rhythmic. (C) Average cosinor amplitude of each organ for each group. Graphs using data from mice whose PER2::LUC expression was rhythmic. (D) Average of core body temperature for each group during 1 day, n = 8 for 35°C, and n = 16 for control and intact groups. All values are expressed as mean ± or + SEM except (A) and (D).

Our data indicated that changes in core body temperature influenced the phase-shift and circadian rhythm; we sought to examine the effect of 35°C ambient room temperature (Fig. 5). All four mice in this group retained the rhythmic PER2::LUC expression and there were no significant differences in phase-shift between the intact (24°C) and 35°C ambient room temperature groups (Fig. 5B). Cosinor amplitude of the kidney significantly decreased after subjecting mice to 35°C or room temperature (Fig. 5C). The core body temperature of these mice at 35°C ambient room temperature elevated rapidly, but also rapidly declined during the treatment (Fig. 5D).

### Temporary temperature pulse at 41°C for 2 h up-regulates *Per* gene expression

**Figure 6:**
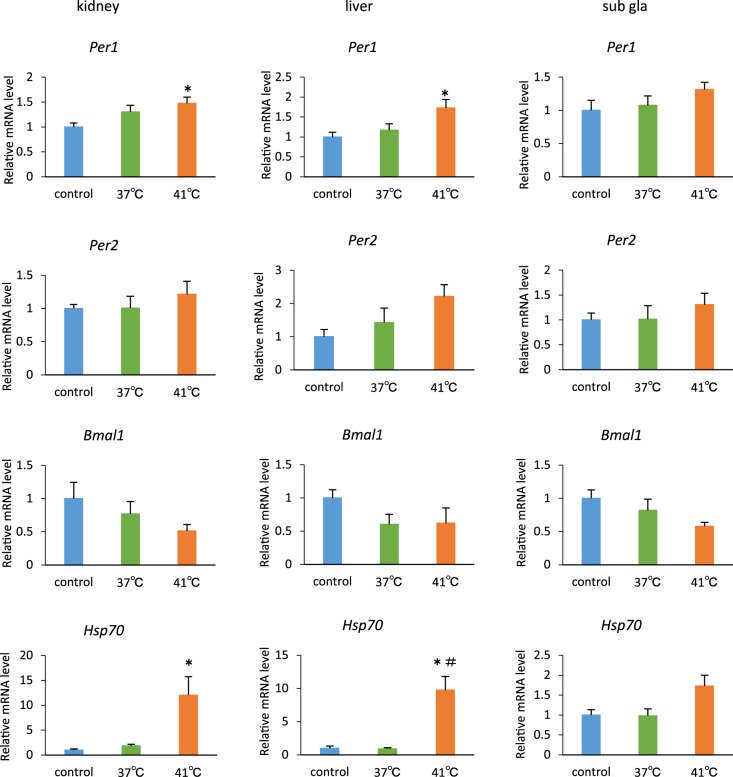
Gene expression, after subjecting mice to temperature pulses in a water bath for 2 h. Mice were exposed to 41°C or 37°C water bath, or a cylindrical sieve as temperature treatment control. The data of control group were set to 1. *p<0.05 versus control; #*p*<0.05 versus 37°C group, by Dunn test, respectively. n = 4 for each group except 41°C group (n = 3) of *Per2* in the liver. All values are expressed as mean + SEM.

In an effort to understand the factors that advance the phase of PER2::LUC expression and circadian rhythms, we investigated the clock gene expression after temporary temperature pulse in a 41°C hot water bath for 2 h. As a result, *Per1* expression was found to be significantly elevated in the kidney and the liver (Fig. 6).

Elevated *Hsp70* levels were observed after subjecting mice to 41°C water bath treatment, indicating that the temperature bath simulated a heat shock effect.

### Glucocorticoid stimulation does not stimulate phase-shift in the peripheral clocks

**Figure 7:**
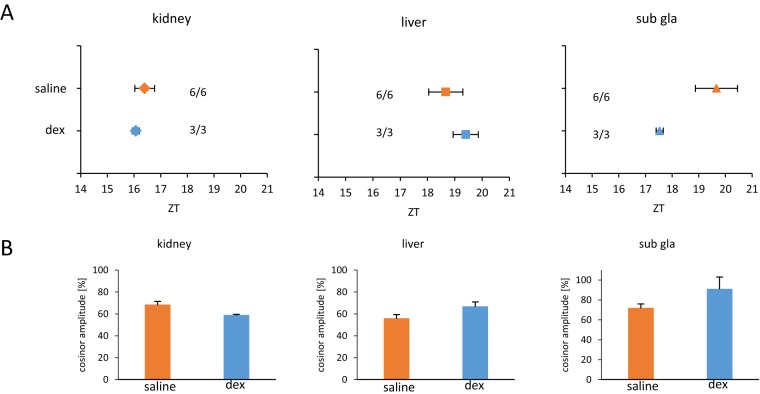
Dexamethasone injection did not shift phase of the peripheral clock. Dexamethasone (dex) was injected to mice at ZT 5 once a day for two continuous days. (A) Average PER2::LUC peak phases of each organ for each group. Numbers next to plots indicate the rates of mice whose PER2::LUC expression was rhythmic. (B) Averaged cosinor amplitude of each organ for each group. Graphs using data from mice whose PER2::LUC expression was rhythmic. All values are expressed as mean ± or + SEM.

Considering that mice received both water and hot stimulation, we were interested to test the effect of stress responses through corticosterone release on the phase-shift of the peripheral clocks. To this end, we treated mice with dexamethasone to understand the exclusive effect of corticosterone on the phase-shift of peripheral clock. Six mice were injected with saline as a control group, and three mice with 10 mg/kg dexamethasone at ZT 5 for two continuous days. Compared to the control group, the dexamethasone group showed no significant phase shifting in any of the organs tested (Fig. 7A). Cosinor amplitude in the organs tested was almost same as the control group (Fig. 7B).

## Discussion

In the present set of experiments, mice exposed to 41°C in a temperature controlled water bath for 2 days during the inactive period resulted in advanced phase of the peripheral clocks in an exposure time (0.5–2 h)-dependent manner. On the other hand, mice exposed to 37°C water bath or control mice placed in a small cylindrical pipe on the sieve at room temperature exhibited a slight phase-advance. The core body temperature of mice maintained at 41°C in the water bath was maintained between 40°C and 41°C during the treatment period, while exposure to 37°C or a small cylindrical pipe were maintained between 38°C and 39°C. Therefore, our data suggest that in the present experimental conditions, a core body temperature change of 40–41°C for at least 2 h is necessary for entraining signals. This hot water-induced phase advance was observed in both female and male mice, suggesting no sexual differences. A change in room temperature from 24°C to 35°C for 2 h increased the core body temperature to 40°C for the first 30 min, but then the core body temperature rapidly declined to 38.5°C, effected by the innate compensation response to ambient high temperature in mice. Thus, the lack of constantly maintained core body temperature increase may be insufficient to cause phase-shift, and the core body temperature fluctuations from the ambient temperature experiments, support this result.

In this experiment, the effect of stress might contribute to the warm water-induced phase-shift. Control mice kept in cylindrical sieve with stainless net made from rough mesh exhibited a small shift of phase-advance in comparison to intact mice. On the other hand, dexamethasone injection at ZT 5 for 2 days did not produce a phase-advance. Taken together, it is suggested that glucocorticoid-release by stress factors such as warm water, cylindrical sieve, and ambient high temperature may not be largely involved in warm water-induced phase-advance. Previously Balsalobre [30] examined the phase-shifts based on timing of dexamethasone injection, and they found a large phase delay after injection around at ZT 11-ZT 18, but no shifts after injection at ZT 5, supporting our current observations.

In *in vitro* cell line experiments, NIH3T3 cells and RAT1 fibroblasts have been reported to show entrained circadian rhythm in temperatures within the physiologic range (36–38.5°C) or routine temperature differences of only 3°C [12], [13]. Furthermore, as observed in our *in vivo* peripheral tissue experiments, temperature-dependent circadian changes comparable to those seen with core body temperature rhythms (36–38°C) are capable of entraining and enhancing the amplitude of the circadian rhythms of peripheral tissues *in vitro* [14]. *In vivo*, in activity rhythm experiments, 60% to 80% of the mice were entrained by ambient temperature cycle (24–32°C) [31]. Although circadian fluctuation of body temperature here was 36.5–38.5°C, and these changes were comparable to previous reports, a core body temperature increase to 38–39°C using a 37°C water bath, or 38.5–40°C at 35°C ambient room temperature, failed to cause any phase-shifts. These results suggest that transient temperature stimulation is not enough as an entraining signal for phase-shift of peripheral clocks under light-dark cycle conditions. There are two types of entraining signals affecting circadian rhythm, parametric and non-parametric stimulation [32]. Parametric temperature stimulation consisting of 12 h high and 12 h low temperature cycle may be effective for entraining signals compared with a non-parametric temperature stimulation, which comprises 1–2 h high or low temperature in a day. In the present study, we adopted a non-parametric stimulation approach because the parametric stimulation does not clarify whether entrainment mediated by temperature cycles is caused directly by temperature or indirectly through the effect of temperature on locomotor and/or feeding activity [31]. Actually motor activity and feeding are well known entraining factors of circadian rhythm [24]–[26]. Thus, this is to our knowledge, the very first study demonstrating the direct entraining effect of temperature on peripheral circadian clocks. Although it is not clear as to why a high core body temperature (40–41°C) is necessary to cause phase shift in the present experiment, one plausible reason is that the current experiment was examined under light-dark cycle conditions, in which the SCN dependent circadian rhythm is active. A peak of phase of the peripheral clock, which is induced by temperature stimulation, which had moved to daytime, may be influenced by SCN and/or nocturnal feeding and therefore may be pulled back to nighttime. Phase-advance by 2 day warm water stimulation was attenuated by 24 h after heating, suggesting this possibility. Thus, it is possible that the warm water (41°C) treatment is too weak to entrain the signal, which is easily influenced by other signals. In the current experiments, it is difficult to rule out the possibility that there is the direct change of luminescence by heat and this direct effect modified estimated clock phase value of the first cycle. In the present experiment, we found that, after the warm water (41°C) stimulation for 2 days, the liver clock rhythm peaked twice. One peak possibly corresponds to the new rhythm and the other to the original rhythm (Fig. S2). Another possible explanation is that each peak was recorded from each subdivision of the liver. However, this explanation can be discarded because we could not find any difference between the subdivisions in the liver (Fig. S3). With regard to the role of the SCN on the warm-water-induced phase advance of the peripheral clock, the possibility that the SCN was entrained by a transient increase in the core body temperature and the possibility of the phase of the peripheral clock thus being indirectly entrained are excluded because the SCN was resistant to temperature entrainment compared to the lung and the pituitary [14]. On the other hand, in the *in vitro* experiment, such a compensatory signal is absent, and circadian rhythm system may exhibit more sensitivity to changing temperature. If compared 3-day warm water stimulation with 2-day warm stimulation, former stimulation became weaker than later one, suggesting that 3-day heating stimulation may be acquired the tolerance response. Among peripheral tissues, the kidney and the liver clock were sensitively impaired by high temperature stimulation compared to the sub gla clocks, because the accuracy of rhythmicity was impaired more in the kidney and the liver after warm water stimulation. Differences in sensitivity of each peripheral clock to entraining signals, such as glucocortisone, and restricted feeding have been previously reported [33].

The resetting of *Per2* expression by temperature pulses in tissue explants has been shown to be blunted in the presence of the heat shock transcription factor (HSF) inhibitor [14] or in HSF1-deficient fibroblasts [15]. Thus, HSF may be involved in the temperature-dependent phase entrainment of circadian clocks [12], [14]. Here in this study, high temperature pulses (41°C for 2 h) transiently increased *Per1*, *Per2*, and *Hsp70* mRNA expression, and decreased *Bmal1* mRNA expression in the peripheral tissues, whereas medium temperature pulses (37°C for 2 h), which resulted in slightly phase-shift did not change the expression levels of these genes. Therefore, the transient change of clock gene expression may trigger the phase-shift.

In summary, we have demonstrated that transient high temperature pulses using water bath during daytime caused phase-advances in mouse peripheral clocks *in vivo*. The present results suggest that hot water bath may affect the phase of peripheral clocks.

## Supporting Information

Figure S1 **Schematic schedule for maintaining mice in the water bath.** (A) Schematic schedule for time-dependent maintenance of mice in a temperature-controlled water bath (B) Schematic schedule for temperature treatment using a water bath and the time schedule for IVIS. (B) In some mice, a 2-day water bath was used and IVIS recording was started 24 h after heating (Day 3).
(EPS)

Figure S2 **Waveform of PER2::LUC expression in each organ in mice that were exposed to temperature pulses in a water bath for 0.5, 1, 1.5, or 2 h (related to** **Fig. 2** **).** All values are expressed as mean ± SEM.
(EPS)

Figure S3 **Analysis of PER2::LUC bioluminescence rhythms in each subdivision of the liver. Representative mice show one peak (A) or two peaks (B) from the whole liver in 2 h group (** **Fig. 2** **).** The left slides represent the actual IVIS images at each time point with the red line compartments to calculate ROI. The right slides represent the waveforms of PER2::LUC bioluminescence from the total or individual ROI. The data for each ROI are shown in a different color.
(EPS)

Figure S4 **Exposing PER2::LUC in male mice to 41°C water bath for 2 days advanced the phase of the peripheral circadian clock.** (A) Waveform of PER2::LUC expression in various tissues. (B) Averaged PER2::LUC peak phases of each organ for each group. The numbers next to the plots indicate the rates of mice whose PER2::LUC expression was rhythmic. *p<0.05, **p<0.01, ***p<0.001 versus control mice by the Student's *t*-test. (C) Averaged cosinor amplitude of each organ for each group. Graphs using data from mice whose PER2::LUC expression was rhythmic. ***p<0.001 versus control mice by the Student's *t*-test. All values are expressed as mean ± or + SEM.
(EPS)

Figure S5 **Waveform of PER2::LUC expression in each organ in mice exposed to temperature pulses in a water bath for 1 day, 2 days, 3 days, and 2 days +24 h (** **Fig. 3** **).** All values are expressed as mean ± SEM. Group “2 days +24. h” represent the mice that were treated with warm water for 2 consecutive days, and the IVIS recording of these mice started 24 after heating.
(EPS)

Figure S6 **Waveform of PER2::LUC expression in each organ in mice exposed to 41°C or 37°C temperatures in a water bath for 2 days (** **Fig. 4** **).** All values are expressed as mean ± SEM.
(EPS)
